# Smile makeover with direct composite veneers: A two-year follow-up report

**DOI:** 10.15171/joddd.2018.023

**Published:** 2018-06-20

**Authors:** Bora Korkut

**Affiliations:** ^1^Department of Restorative Dentistry, Faculty of Dentistry, Marmara University, Istanbul, Turkey

**Keywords:** Direct veneer, aesthetic restorations, emulating nature

## Abstract

Direct composite veneers have gained an important role in dental clinical applications following recently developed materials and techniques in adhesive and restorative dentistry. Direct application on prepared tooth surfaces or even without any preparation, with an adhesive agent and a composite resin material in a single visit is the main procedure as well as the advantage of these restorations. The main aim of this direct procedure is to create minimally invasive and long lasting restorations. As the dental materials and techniques develop, the clinicians also had the chance to mimic the natural dental tissues to create very natural alike restorations in a single appointment. But like all the other dental procedures, direct composite veneers have some indications and contra-indications. These musts have to be understood very well by the clinician before the treatment planning. But this way direct composite veneers would no longer named as 'day savior fillings' and called as minimally invasive, functional and long lasting 'direct aesthetic restorations' that perfectly emulate the natural dental tissues. This article discusses the necessities of direct composite veneers for the ultimate success and illustrates how to perform a minimally invasive, long lasting, functional and natural alike smile makeover with these restorations in a single visit.

## Introduction

Veneers with direct resins are one of the common treatment options for clinical applications following the developments in adhesive and restorative dentistry in recent years. These restorations are applied on prepared tooth surfaces or even without any preparation, with an adhesive agent and a composite resin material directly in a single visit in the dental clinic.^1^ If done properly, the aesthetic outcomes of direct composite veneers are very satisfactory in addition to superior optical and physical properties.^2^ In recent history these restorations were thought to be temporary alternatives to indirect ceramic veneers; however, they are no longer named 'day savior fillings' today. These restorations are called minimally invasive, functional and long-lasting 'direct aesthetic restorations' that perfectly emulate natural dental tissues even in anterior area.^3,4^ Discolorations of teeth or restorations, dental malformations or mal-positions, diastemas, crown fractures and abrasive or erosive defects are some examples of up-to-date indications of direct composite veneers.^1^ Enamel hypoplasia is a developmental malformation generally resulting in poor aesthetics, tooth sensitivity, malocclusion and predisposition to dental caries.^5^ Direct composite veneer restorations where the whole labial surface is covered with resin, are good treatment options in such cases.,^6^

This case report describes a step-by-step one-day smile makeover with direct composite veneers on maxillary incisors and canines with severe enamel hypoplasia. Two-year clinical follow-up of the restorations is evaluated and the success is discussed.

## Methods

**Figure 1:**
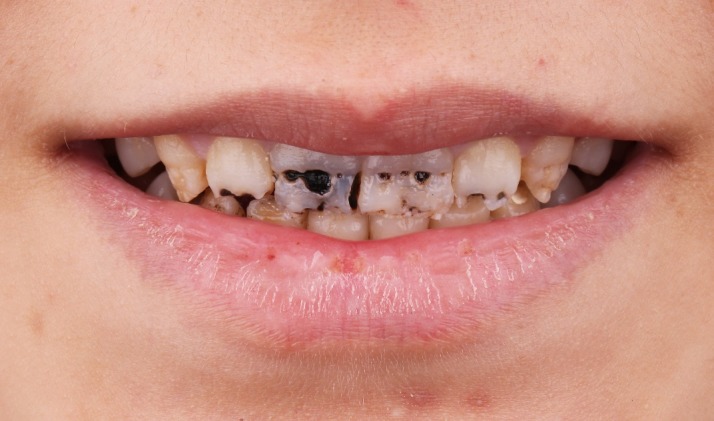
Initial

A 14-year-old female patient applied to the clinic with aesthetic complaints and smile make-over demand. Clinical examination revealed multiple defects generally combined with carious lesions on labial surfaces of crowns in both jaws. Some defects were local and some were diffuse on the labial surfaces of maxillary anterior teeth mainly. The patient reported that the defects were due to severe enamel hypoplasia (Figure 1). Oral hygiene was good and soft tissues were healthy. The patient did not have any other symptoms. In addition, there was no periapical lesion in radiographic examinations; therefore, all the teeth were considered as vital. The patient also revealed that her economic condition was not very good. Considering all the examinations and the data collected, a smile make-over with direct aesthetic composite veneers primarily on maxillary incisors and canines in a single appointment was determined as the treatment plan. Minimally invasive preparations, good rubber dam isolation and a silicone index guidance were also determined as necessities for this case.

**Figure 2:**
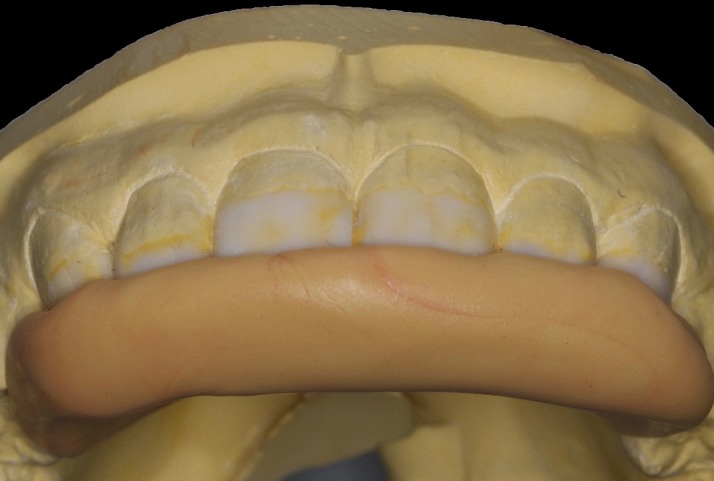
Silicone impression

**Figure 3:**
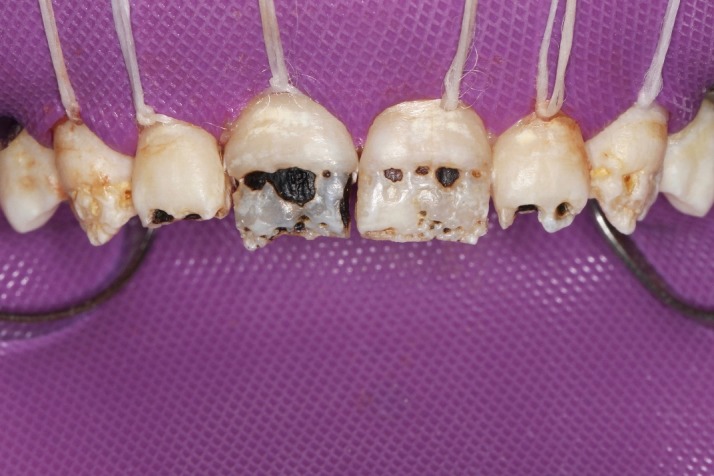
Isolation

**Figure 4:**
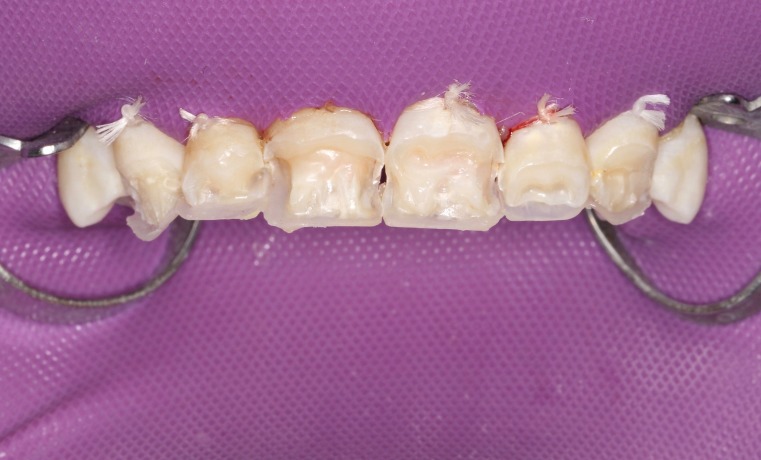
Preparations

**Figure 5:**
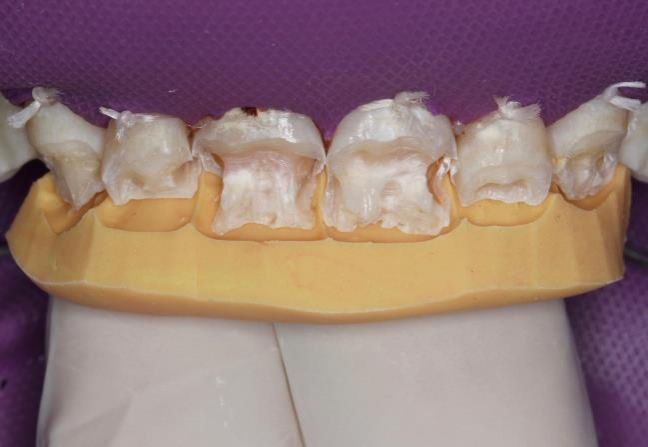
Silicone index trial

**Figure 6:**
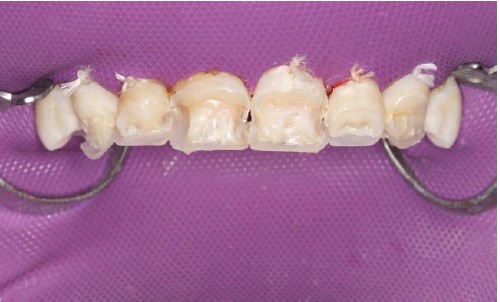
Palatal walls

**Figure 7:**
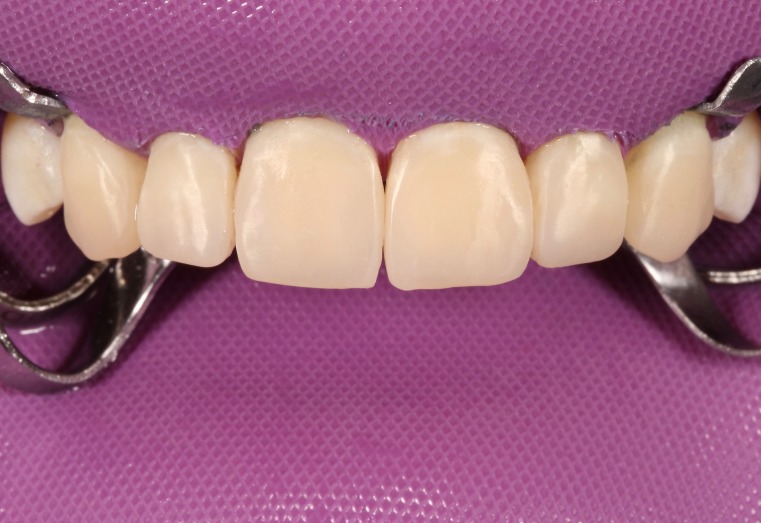
Final before polishing

**Figure 8:**
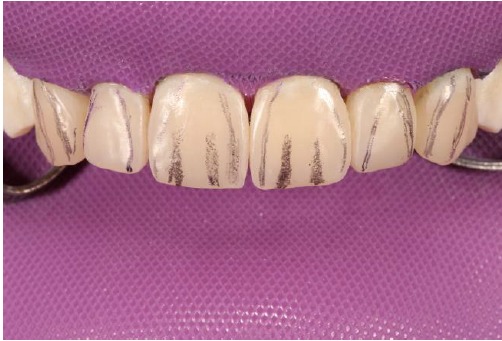
Provisional macro surface texture

**Figure 9:**
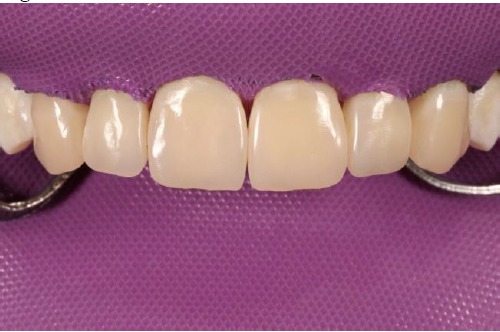
Final after polishing

**Figure 10:**
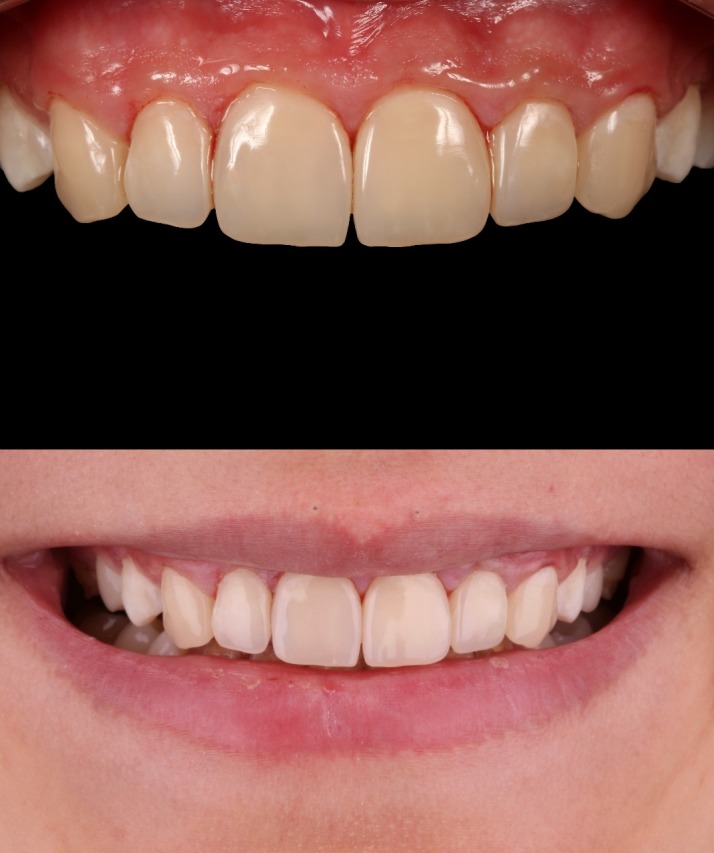
Figure 10. Final restorations (Just after the restora-tions).

First, flat-surfaced round composite button samples in different shades were placed on the middle third of the maxillary right lateral incisor and a cross-polarization macro dental photography was taken. The most proper shades were selected on the photo as A1B (body shade) and OcE (translucent shade; Estelite Asteria, Tokuyama, Japan). Then temporary wax-up restorations were carried out on the cast model of the patient's upper jaw and silicone impression was taken to create the palatal silicone index (Figure 2). The dimensions of the temporary wax-up restorations were adjusted according to the upper and lower lip positions of the patient in resting and smile conditions. The teeth were isolated with rubber dam and retracted with simple dental floss (Figure 3). Minimally invasive preparations rather than full veneer preparations were performed on maxillary central incisors and canines (Figure 4). The depth of the preparations was limited in enamel tissue as much as possible. The silicone index was tried on the prepared teeth (Figure 5). The layering of the restorations was carried out one by one for each tooth and the adjacent teeth were isolated with a Teflon tape. Then 37% orthophosphoric acid was applied selectively, washed and dried, and a universal adhesive agent was applied (G-Premio Bond, GC, Japan). A very thin layer of low-value OcE shade resin was applied on the silicone index. Then the index was placed on the teeth and polymerized to create palatal walls (Figure 6). To emulate the natural dental tissues, dentin layering was carried out with medium value A2B shade and surface enamel layering was carried out with low-value OcE shade by using a composite brush and wetting resin (Composite Primer, GC, Japan; Figure 7). Before polishing, oxygen inhibition layer was eliminated by using glycerin gel (Air Barrier, GC, Japan). Provisional surface macro-morphology was drawn on the surface of each tooth and surface macro-texture was created by using a yellow-banded diamond bur at low speed and in dry conditions (Figure 8). Interdental reductions were made by using a #12 blade gently. Interdental polishing was carried out with interdental strips in different grains from coarse to fine (Epitex, GC, Japan). Marginal roundings and line angles were created by using polishing discs in different grains from coarse to fine (SofLex, 3M, Japan). Coarse polishing of the surfaces was carried out with a spiral, rubber polishing disc (Twist Dia, Kuraray, Japan). Surface micro-texture was created for each tooth with a red-banded diamond bur at very low speed, in one direction and under dry conditions (Figure 9). The rubber dam was removed and occlusal relations were controlled (Figures 10). The whole smile makeover procedure took 3.5 hours of chair-time. Oral hygiene instructions were given and the patient was called for recall visits at 3-, 6- and 12-month and yearly intervals thereafter.

**Figure 11:**
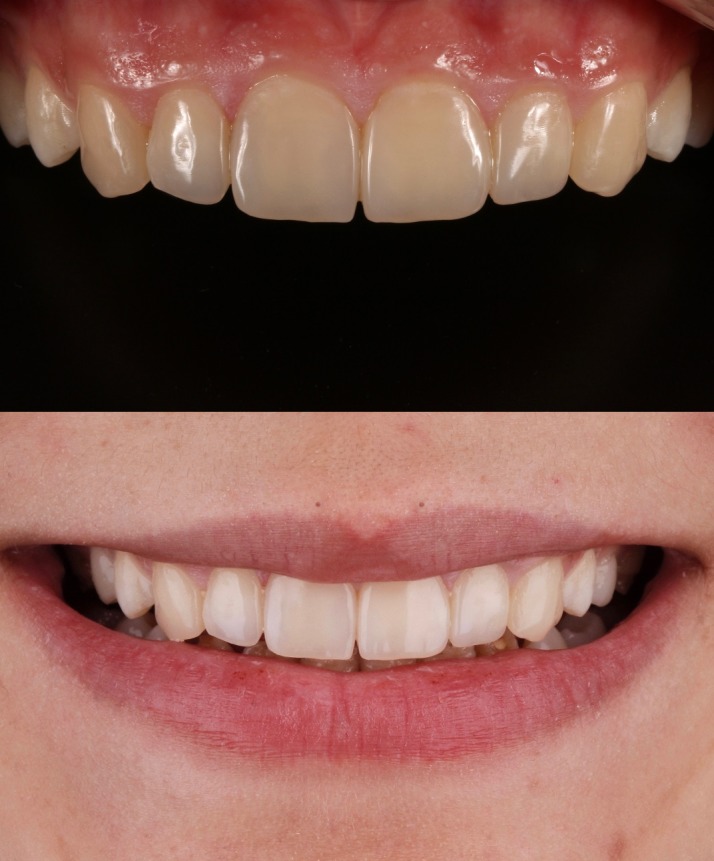
Figure 11. Final restorations (Two years follow-up)

In the two-year follow-up, the restorations were stable and fully functional. No fractures or cracks were detected on the restorations (Figures 11). The patient also reported that she was very happy with the aesthetic appearance as well as the durability of the restorations. Even in cross-polarized and high-contrast dental control photography, no dis-coloration or demarcation line was detected. Only the surface micro-texture of central incisors and canines created with diamond bur were barely observable in one-year and two-year follow-ups. However, this disparity could only be seen in macro dental photography with angled flash light. Oral hygiene was satisfactory and gingival tissues were considered as healthy. In addition, no peri-apical lesion was detected in radiographic control. Based on all these data, the treatment was consid-ered as successful in the two-year recall.

## Discussion

Enamel hypoplasia, in other words chronic enamel aplasia in primary dentition, is a developmental disease related mainly to some systemic factors such as birth trauma, infections, nutritional disorders, metabolic diseases and tetracycline or fluoride exposure. It generally results in poor aesthetics, tooth sensitivity, malocclusion and predisposition to dental caries.^5^ Direct composite veneer restoration, as a minimally invasive method with long-lasting functional and aesthetic success, is one of the most preferred treatment alternatives for restoration of anterior teeth, especially in young patients.^2,6^

In this case report, a smile makeover of a young patient suffering from enamel hypoplasia was performed with direct composite veneers in a single appointment. Shade selection of the composite resin was carried out before isolating the teeth to avoid the color change after dehydration.^7^ Cross-polarization dental photography with composite button technique was used as the most up-to-date method for shade selection.^7-9^ It has been reported that with this technique the light reflections on the tooth surface, which might misdirect the clinician in shade selection, are eliminated.^8-10^ The surface of the composite button samples were also flattened to avoid the unfavorable reflections. As the most predictable method, a wax-up-based silicone index was used for composite layering. The clinician can take advantage of ultimate control of items like the dimension of palatal and incisal walls, interproximal emergence profiles, surface macro-micro textures and chromatic characteristics when using a wax-up-based silicone index.^11^ The dimensions of the temporary wax-up restorations were adjusted according to the upper and lower lip positions under resting and smile conditions to avoid devastating occlusal forces. The preparation of composite veneer is more conservative compared to ceramic veneer.^1,2^ As the malformed maxillary incisors and canines had carious lesions in various locations and depths, the preparations were done in a minimally invasive manner to avoid excessive dental tissue removal. The depth of the preparations were limited in enamel tissue as much as possible according to the fact that the more the enamel tissue remains the better the adhesion is.^12^ Selective etching method was used to achieve better enamel adhesion without dentin hypersensitivity.^12^ As a necessity in all the adhesive procedures, a good rubber dam isolation was provided.^13^ In order to eliminate the oxygen-inhibited layer to create a better-polymerized and harder surface, glycerin gel was used. It is a fact that this action positively affects the surface hardness and polishability as well as the color stability of composite resin restorations.^14-17^ One of the most up-to-date and effective polishing materials, diamond particles-embedded spiral rubber polishing discs (Clearfil Twist Dia, Kuraray, Japan) were used for surface polishing.^18-20^ According to the one-year and two-year follow-up reports, although the restorations were not re-polished, they maintained their high-level surface roughness and glow. In addition, no discolorations or demarcation lines were detected in any part of the restorations. This might be related to the positive effect of the polishing system used in surface polishing as well as the effect of elimination of the inhibition layer.^16,18-20^ The physical properties of the composite resin used in this case (Estelite Asteria, Tokuyama, Japan) might have probably affected this positive result, too.^15,21,22^ The physical properties are reinforced with the unique spherical shape of the fillers to have better surface hardness, polishability and color stability.^21^ Furthermore, the interdental polishing probably positively affected the resistance to discoloration since the discoloration usually starts from this area.^18^ The polishing discs were only used for marginal rounding and line angle creation in this case and not for surface polishing. The reason is that even the one with ultra-fine grains removes restoration material from the surface, compromising the surface micro- and macro-textures. In the case presented, the final surface was polished without removing the textures by using only a fine-grained spiral rubber polishing disc in dry conditions. This spiral disc with unique feature is also produced by two different companies other than the one used in this report and all are very effective in surface polishing in a very short chair-time. Starting from one-year follow-up, the surface micro-textures of central incisors and canines were barely observed. The loss might be related to the erosive or abrasive nature of the patient's diet and brushing style or to the surface roughness of the restoration.^19^ However, it was insignificant for the aesthetic appearance from speaking distance.

In recent years direct composite veneers have been compared to indirect ceramic veneers and declared as weak in relation to resistance to fractures and discolorations. However, the fact that everyone misses is whether these direct resin restorations were done correctly or not? Direct composite veneers have indications and contraindications like almost all other dental procedures which can be listed as proper occlusion, lateral and protrusive movements,^1,4^ correct shade analysis,^8-10^ effective isolation,^13^ good adhesion,^12,13^ effective polishing,^18-20^ eliminating oxygen inhibition layer,^16^ frequent recalls,^11^ high-quality materials^17^ and clinical experience.^12^ These are pieces of a whole and if one fails, then the whole restoration fails. If the direct composite veneers are carried out by observing the rules, the success rate will definitely increase.

## Conclusions

Under the conditions of this two-year follow-up case report, direct composite veneers, as single-visit, minimally invasive, discoloration-resistant, long-lasting and aesthetic restorations, are one of the most promising treatment options even for the smile makeover cases in clinical applications if done by the rules. Recently called 'day savior fillings', they should be called long-lasting 'direct aesthetic restorations' as they deserve.

## Acknowledgments

The head of department provided general support.

## Authors’ contributions

Dr. Bora Korkut developed the original idea and the protocol, abstracted and analyzed the data and was the grantor.

## Funding

This study was supported in part by the Faculty of Dentistry, Marmara University.

## Competing interests

The author declare no competing interests with regards to the authorship and/or publication of this article.

## Ethics approval

The reported patient has given wirtten informed consent for the publication of this paper.
